# Work-related musculoskeletal injuries in formal caregivers of Portuguese rest home of elderly

**DOI:** 10.1590/1806-9282.20241034

**Published:** 2025-03-31

**Authors:** Beatriz Minghelli, Andreia Inácio Guerreiro, Marketa Pinho, Diogo Manuel Rafael Gomes, Rita Sofia Ribeiro Antunes, Chloé Rafaela Colaço Nunes

**Affiliations:** 1 INSIGHT – Piaget Research Center for Ecological Human Development – Lisboa, Portugal.; 2 Instituto Piaget de Silves – Silves, Portugal.

**Keywords:** Epidemiology, Caregivers, Cross-sectional study, Injuries, Risk factors, Occupational illnesse

## Abstract

**BACKGROUND:**

The formal caregiver is the professional who provides healthcare to the elderly person in carrying out tasks that they are unable to carry out independently with personal care, daily activities, and general well-being. These professionals are exposed to a variety of factors that can contribute to the development of work-related musculoskeletal disorders.

**OBJECTIVE:**

The aim of this study was to determine the epidemiology of work-related musculoskeletal disorders in formal caregivers of rest homes of the elderly in the central-southern regions of Portugal and the associated risk factors.

**METHODS:**

The sample consisted of 251 formal caregivers from Santas Casas da Misericórdia, of which 228 (90.8%) were female, aged between 21 and 65 years old. The measuring instrument consisted of a digital questionnaire.

**RESULTS:**

The majority of caregivers (186, or 74%) reported experiencing work-related musculoskeletal disorders over the past 12 months, accounting for 374 reported cases. Specifically, 143 (71.9%) caregivers encountered 255 work-related musculoskeletal disorders in this period. The overall injury proportion was 0.72, and the injury rate was 0.12 injuries/1,000 h of work. The most common types of work-related musculoskeletal disorders included low back pain (64 cases, 26.8%), non-specific pain (52 cases, 21.8%), and tendinopathy (51 cases, 21.3%). The primary locations of these disorders were lumbar spine (82 cases, 34.3%), shoulder (42 cases, 17.6%), and cervical spine (26 cases, 10.9%). The leading mechanisms of injury were transfers (164 cases, 35.6%) and repetitive movements (139 cases, 30.2%). Additionally, the female formal caregiver had 3.8 times (95%CI 1.1–12.3; p=0.029) the likelihood of developing work-related musculoskeletal disorders compared to their male counterparts.

**CONCLUSION:**

Data from this study revealed a high work-related musculoskeletal disorders presence in formal caregivers, with women presenting a higher risk of developing injuries. The development of prevention programs is necessary to improve work performance and the health of these professionals.

## INTRODUCTION

In the demographic domain, the most recent projections indicate that in 2070 Portugal will have a greater increase in the number of elderly people, with people over 80 years old accounting for 16.1% of the population^1^. In response to this trend, a set of structures was created in Portugal at the health and social level (homes, day centers, home support services) aimed at monitoring this age range, which allows the family greater support and security in times of unavailability^2^.

The formal caregiver is a professional hired and paid to provide health care to the elderly in carrying out tasks that they are unable to perform independently and that are essential for their well-being and quality of life, care that can include nutrition, personal hygiene, education, culture, daily activities, and general well-being, who can work in hospitals, homes, and community institutions^3,4^.

The health professionals’ activity involves exposure to a variety of risk factors that can contribute to the appearance and development of work-related musculoskeletal disorders (WMSDs)^5^. It is estimated that employees in nursing and personal care homes suffer approximately 200,000 work-related injuries and illnesses per year^6^.

Several physical risk factors may be associated with WMSD, such as adopting posture and working in inappropriate positions, heavy physical work, lifting loads and repetitive movements, lack of rest breaks, and demanding work and stress^7,8^.

Given the type of work carried out by formal caregivers, the evidence seems to link this population with a high risk of WMSDs; however, studies on this topic are still scarce, especially in Portugal. The physical demand of the role of formal caregiver represents a challenge in the daily lives of these workers. Physical exhaustion, poor postures adopted, and associated repetitive movements applied to a population that is mostly dependent and not always easy to handle can lead to the occurrence of WMSDs in these workers^9–11^.

This study aimed to determine the epidemiology of WMSDs in formal caregivers of rest homes of the elderly in the central-southern regions of Portugal and to determine the associated risk factors.

## METHODOLOGY

It was a descriptive-correlational cross-sectional study approved by KinesioLab—Research Unit in Human Movement. Formal caregivers were informed of the objectives and nature of the study and that they could withdraw from the study at any time without any harm, and the anonymity of individual responses was guaranteed.

### Population

The population consisted of formal caregivers from Santas Casas da Misericórdia do Algarve and Alentejano Coast who had performed this role for at least 3 months. The inclusion criteria involved formal caregivers of any gender, aged 18 years or over, and those who agreed to participate in the study.

The sample calculation took into account the number of formal caregivers who worked in Santa Casa da Misericórdia and the number of our population was 392 formal caregivers. Since there are few studies on this topic, we estimate a prevalence of 50% of WMSDs, a confidence interval of 95%, and an error margin of 5%, and we set an n number of 195 formal caregivers as the representative sample.

### Measuring instrument

The measuring instrument consisted of a questionnaire, in digital format, prepared by the researchers themselves. The questionnaire was evaluated by a panel of experts made up of a physiotherapist with a PhD in epidemiology, a nurse with a PhD in public health, and a formal caregiver with more than 15 years of experience in the activity. In addition, a pre-test was carried out on 10 formal caregivers from Santa Casa da Misericórdia of Silves.

The questionnaire contains questions about the socio-demographic characterization of the population, work characteristics, and the occurrence of WMSD. Only the caregiver who had a WMSD in the last 12 months continued to fill out the questionnaire. Workers who had more than three WMSDs could only describe the three WMSDs classified as the most serious.

A WMSD was defined as any condition or symptom that occurred as a result of the role of the formal caregiver and that had at least one of the following consequences: having to stop working as a formal caregiver for at least one day; if you did not have to interrupt the activity, but had to change the activity (less number of hours in the role, less ability to perform certain gestures or maneuvers), sought advice or treatment from healthcare professionals to resolve that condition or symptom.

### Data analysis

The software used to perform the statistical analysis of the data was the Statistical Package for Social Sciences (SPSS), version 28.0.

In the first approach, descriptive statistics were performed. The binary logistic regressions (enter methods) were applied to test the influence of the variables analyzed in this study with the WMSD presence. A final multivariate model was developed, using the forward likelihood method, and its validity, quality of fitting, and predictive capacity were assessed by Omnibus and Hosmer-Lemeshow tests and the Nagelkerke correlation coefficient. The level of statistical significance was established at 0.05.

## RESULTS

The sample consisted of 251 formal caregivers, exceeding 28% of the expected sample size. The majority were female (228; 90.8%) and only 23 (9.2%) workers were male, aged between 21 and 65 years (45.84±10.78).

Table 1 presents the variables on the professional characteristics of formal caregivers, indicating the relative and absolute frequencies. The weekly working hours varied between 15 and 48 h (39.8±2.9 h) and the break time was between 15 and 150 min (2.5 h) (64.9±23.3 min).

**Table 1:** Relative and absolute frequencies of professionals’ characteristics.

Professionals’ characteristics		n	%
Work bond	Full-time	249	99.2
	Part-time	2	0.8
Career years	3–6 months	33	13.2
	7 months until 1 year	19	7.6
	1 until 3 years	51	20.3
	3 until 5 years	27	10.8
	≥6 years	121	48.2
Shifts	Fixed	138	55.0
	Rotary	113	45.0
Position that works the most	Standing	249	99.2
	Seated	2	0.8
Other professional activity	No	227	90.4

Regarding the practice of physical exercise, with a minimum frequency of twice a week, 80 (31.9%) caregivers responded that they practiced it.

During the entire work practice, 186 (74.1%) caregivers suffered an injury, with 82 (44.1%) caregivers reporting suffering 1 WMSD, 52 (28%) 2 WMSDs, 20 (10.8%) suffering 3 WMSDs, and 32 (17.2%) suffering 4 or more WMSDs, totaling 374 WMSDs. At the moment of evaluation, 119 (47.4%) caregivers were injured. In the 6-month period, 86 (39.4%) caregivers suffered injuries. In the last 12-month period, 143 (71.9%) caregivers reported injuries, with 68 (47.6%) caregivers reporting suffering 1 WMSD, 42 (29.4%) suffering 2 WMSDs, 29 (20.3%) suffering 3 WMSDs, and 4 (2.8%) suffering 4 or more WMSDs, totaling 255 WMSDs. In total, 20 (9.2%) workers reported that they were prevented from carrying out their professional activity in the last 6 months because of the WMSD.

The value of injury proportion was 0.72 (95%CI 0.17–0.28) and the injury rate was 0.12 injuries/1,000 h of work. The average number of injuries per formal caregiver was 1.28, and the average of injuries per injured formal caregiver was 1.78.

Table 2 shows the type and anatomical location of the WMSDs in the analyzed sample. The number of total WMSDs shown is less than the total number of WMSDs in the 12-month period because only a maximum of 3 WMSDs were classified per formal caregiver.

**Table 2:** Type, location, mechanism of work-related musculoskeletal disorders, and treatments.

Types of WMSDs	Total	Location of WMSDs	n	Location of WMSDs	n
Non-specific pain	52 (21.8%)	Thorax/chest/ribs	2	Hand and fingers	1
		Lumbar spine	3	Pelvis	3
		Shoulder	13	Thigh	3
		Arm	5	Knee	6
		Forearm	1	Leg	4
		Elbow	1	Foot and fingers	5
		Wrist	5	All	52
Neck pain	25 (10.5%)	Cervical spine	25		
		All			
Low back pain	64 (26.8%)	Lumbar spine	64		
		All			
Tendinopathy	51 (21.3%)	Shoulder	25	Knee	2
		Elbow	11	Ankle	1
		Wrist	12	All	51
Disc herniation	15 (6.3%)	Cervical spine	1		
		Lumbar spine	14		
		All	15		
Arthrosis/Osteoarthritis	9 (3.8%)	Lumbar spine	1	Hand and fingers	2
		Knee	6	All	9
Sprain	6 (2.5%)	Knee	4	All	6
		Ankle	2		
Carpal tunnel syndrome	5 (2.1%)	Wrist	5		
		All			
Fracture	3 (1.3%)	Thorax/Chest/ribs	2	All	3
		Pelvis	1		
Epicondylitis	2 (0.8%)	Elbow	2		
		All			
Quervain's disease	1 (0.4%)	Hand and fingers	1		
		All			
Others	6 (2.5%)	Shoulder	4	All	6
		Foot and fingers	2		
**Location of WMSDs**	**n (%)**	**Location of WMSDs**		**n (%)**	
Cervical spine	26 (10.9%)	Pelvis		4 (1.7%)	
Thorax/chest/ribs	4 (1.7%)	Thigh		3 (1.3%)	
Lumbar spine	82 (34.3%)	Knee		18 (7.5%)	
Shoulder	42 (17.6%)	Leg		4 (1.7%)	
Arm	5 (2.1%)	Ankle		3 (1.3%)	
Forearm	1 (0.4%)	Foot and fingers		7 (2.9%)	
Elbow	14 (5.9%)				
Wrist	22 (9.2%)				
Hand and fingers	4 (1.7%)				

WMSDs: work-related musculoskeletal disorders.

The injury mechanisms mentioned were transfers (164; 35.6%), repetitive movements (139; 30.2%), support in daily life activities (97; 21%), uprisings (25; 5.4%), falls (22; 4.8%), and others (14; 3%).

Table 3 shows the relationship between the presence of WMSDs and with non-modifiable factors and work characteristics over the past 12-month period. The final model was considered mathematically valid with a relatively good predictive capacity (Omnibus, Hosmer-Lemeshow, and Nagelkerke tests: p=0.004; p=0.986; and R^2^=0.077).

**Table 3:** Relationship between the event of the presence of work-related musculoskeletal disorder and variables about non-modifiable sample factors and work characteristics of formal caregiver.

Variables	OddsRatio_crude_ (95%CI); p-value	OddsRatio_adjusted_ (95%CI); p-value
Sex (male*) female	4.6 (1.4–14.7); 0.010	3.8 (1.1–12.3); 0.029
Age group (until 49 years old*) ≥50 years old	2.2 (1.2–4.2); 0.016	1.9 (1.0–3.8); 0.560
Career years (until 5 years*) ≥6 years	1.9 (0.9–3.4); 0.052	–
Shifts (fixed*) rotary	1.9 (1.0–3.6); 0.040	–
Break time (until 1 h*) ≥1 h	1.2 (0.6–2.2); 0.647	–
Weekly workload (≥40 hs*) <40 h	1.3 (0.6–2.9); 0.568	–
Physical exercise practice (ye*) no	1.6 (0.8–3.1); 0.159	–

* Class reference.

CI: confidence interval.

It was found that female formal caregivers had a 4.6 times higher probability of developing WMSDs than male workers, as well as the older caregiver (age equal to or more than 50 years old) who presented a 2.2 times higher probability of developing WMSDs compared with the younger ones. Caregivers who worked more years (equal to or more than 6 years) and also those who worked rotary shifts showed 1.9 times higher chances of developing a WMSD. Using the forward likelihood method, only the sex and age group variables remained in the equation, but only the sex variable achieved statistical significance (p=0.029) presenting a 3.8 times higher probability of developing WMSDs in female workers.

## DISCUSSION

Data from this study revealed a high prevalence of WMSD in the analyzed sample. Zaalberg et al.^12^ evaluated 78 caregivers of patients aged 70 years or older visiting the Emergency Department in the Netherlands and verified that 39% of caregivers experienced a high burden, and Maia Junior et al.^13^ evaluated 40 formal and informal caregivers of elderly people at home in Brazil and found that 97.5% of caregivers presented musculoskeletal symptoms.

The high prevalence of WMSDs observed in this sample can be explained by their lack of training for the exercise of such a specific profession; it can also be associated with the lack of physical preparation of workers, as this is a job that requires a high level of physical demand, and the majority of workers in our sample did not practice any type of physical exercise regularly.

The types of WMSDs most frequently referred to in formal care in our sample were low back pain (27%), non-specific pain (22%), and tendinopathy (21%). In these two types of injuries, the most frequent locations were the shoulders. Tomioka and Matsunaga^14^ carried out an interview survey with managers and caregivers of nursing homes. One of the managers mentioned that 80% of his workers complain of low back and upper limb pain.

Low back pain can be explained by tasks that require great physical effort on the part of caregivers, such as transfers; the lack of materials with ergonomic conditions, such as beds and chairs; the lack of a good muscular structure of the caregiver himself; and also the lack of knowledge and/or application of correct techniques for lifting loads, transferring users, and appropriate positioning to carry out activities.

The high presence of non-specific, undiagnosed pain can be explained by the difficulties in resorting to a health professional, whether due to waiting time, the cost of the consultation, the time spent to go to the consultation, or the belief that it is a situation passenger who does not require medical care.

The main WMSD mechanism reported by formal caregivers in this study was precisely the transfer of users. Figueiredo et al.^15^ revealed that transfers were considered the factor that most affected physical health in the opinion of formal caregivers (69%).

The second WMSD mechanism most mentioned by caregivers was repetitive movements. Rhode and Rhode^16^ identified occupational risk factors for tendon dysfunction in the shoulder and observed that repetitive movements maintained at 60° of flexion or abduction, such as helping with activities of daily living (hygiene or transfers), were associated with this type of injury.

The anatomical sites most affected by WMSDs were the lumbar spine (34%), followed by the shoulder (18%) and cervical spine (11%). Similar data were obtained in the study by Bazo and Gimenez^11^, which found a prevalence of 33.9% of injuries located in the lumbar spine, followed by the cervical spine with 26.8%.

The greater presence of injuries in women can be explained by the clothing of estrogen, which promotes an effect on the structure and function of musculoskeletal tissues, such as muscles, tendons, and ligaments, promoting an improvement in muscle mass and strength, increasing the collagen content of connective tissues, reducing stiffness, and directly affecting performance and injury rates^17^. Vega-Vélez et al.^18^ verified some factors associated with musculoskeletal injuries in caregivers, these being a woman.

This study had some limitations, such as the WMSD information being self-reported and not diagnosed by a health professional and the memory bias. Future studies are suggested involving a national representative sample.

## CONCLUSION

Our data revealed a high WMSD presence in formal caregivers analyzed in this study, with the most common WMSD types being low back pain, non-specific pain, and tendinopathy, located in the lumbar spine, shoulder, and cervical spine anatomical body regions, and the main mechanism of WMSD was transfers and repetitive movements. The women present a higher risk of developing injuries.
